# Urinary Retention and Body Lateropulsion by Lateral Medullary Infarction: A Case Report

**DOI:** 10.7759/cureus.78612

**Published:** 2025-02-06

**Authors:** Akiho Maeda, Koji Hayashi, Mamiko Sato, Asuka Suzuki, Yuka Nakaya, Hiroaki Maeda, Yasutaka Kobayashi

**Affiliations:** 1 Department of Rehabilitation Medicine, Fukui General Hospital, Fukui, JPN; 2 Graduate School of Health Science, Fukui Health Science University, Fukui, JPN

**Keywords:** body lateropulsion, lateral medullary syndrome (wallenberg syndrome), lateropulsion, stroke, urinary retention (ur), wallenberg syndrome

## Abstract

We describe a case of lateral medullary infarction (LMI) presenting with both body lateropulsion (BL) and urinary retention (UR). A 29-year-old Filipino male with a history of untreated hypertension, dyslipidemia, hyperuricemia, and obesity presented with acute onset of rotatory vertigo, gait disturbance, and left-sided sensory loss. Initial examination revealed horizontal nystagmus to the left, right-sided facial sensory loss, and left-sided limb sensory loss, without cranial nerve deficits or UR. Brain magnetic resonance imaging showed hyperintensities in the right dorsolateral medulla oblongata, leading to a diagnosis of LMI. Antiplatelet therapy and rehabilitation were initiated. Subsequently, the patient developed dysphagia, UR requiring intermittent catheterization, and right-sided Horner’s syndrome. He also reported right-sided BL during gait training. Following medical and rehabilitative management, symptoms gradually improved, with the resolution of vertigo and UR by day 20, improved BL by day 40, and independent ambulation by day 70, despite persistent slight sensory disturbance. In this case report, we discuss the causes of BL and UR in LMI, comparing them with previous cases.

## Introduction

Lateral medullary infarction (LMI), also known as Wallenberg syndrome, is a clinical condition resulting from an infarction in the territory of the posterior inferior cerebellar artery and its branches [1,2]. This syndrome represents a well-documented vascular disorder of the brainstem, characterized by a wide spectrum of clinical symptoms. Common manifestations include vertigo, nausea, vomiting, headache, ipsilateral Horner syndrome, skew deviation of the eyes, nystagmus, dysphagia, dysarthria, hoarseness, ipsilateral diminished gag reflex, cerebellar ataxia, and crossed sensory disturbances, with ipsilateral facial and contralateral body hypoesthesia as defining features [1,2].

Although many of these symptoms are frequently encountered, some manifestations are less common yet clinically significant. For instance, ipsilateral axial lateropulsion (also known as body lateropulsion, BL), reported in a subset of patients and reflecting unique vestibulospinal involvement [3-5], is characterized by an involuntary propensity to deviate toward one side of the body, without any evident weakness or impairment of voluntary motor control [4,5]. Another rare but notable complication is urinary retention (UR), which underscores the potential for LMI to impact autonomic pathways [6]. These rare symptoms highlight the need for clinicians to maintain a high index of suspicion when diagnosing and managing LMI.

In 2024, we reported the first documented case of LMI presenting with the simultaneous occurrence of BL and UR caused by atonic bladder, a rare combination that expanded our understanding of the clinical spectrum of this condition [7]. This case demonstrated the potential for LMI to affect multiple systems beyond the typical neurological manifestations. Building upon this, we now present a second case of LMI with concurrent BL and UR. This report not only underscores the diverse presentations of LMI but also reinforces the importance of recognizing rare complications to ensure accurate diagnosis and tailored management.

## Case presentation

**Figure 1:**
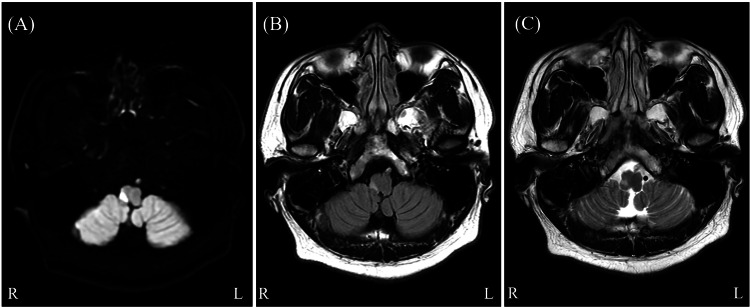
Brain magnetic resonance imaging (MRI) results. The brain MRI reveals hyperintensity in the right lateral medulla oblongata. (A) Diffusion-weighted imaging. (B) T2 fluid-attenuation inversion recovery imaging. (C) T2-weighted imaging.

A 29-year-old Filipino male, with a history of untreated hypertension, dyslipidemia, hyperuricemia (all noted since his 20s), and obesity, presented with the sudden onset of rotatory vertigo in March 2024. The vertigo was accompanied by gait disturbance, nausea, a single episode of vomiting, hypoesthesia to temperature, and pain in the left upper and lower limbs. Despite the persistence of symptoms, he initially visited a local clinic and was subsequently referred to our hospital. The vital signs on admission were as follows: height, 162 cm; weight, 80.0 kg; body temperature, 36.6°C; pulse rate, 75 beats/minute; and blood pressure, 130/76 mmHg. Neurological examination revealed high-frequency horizontal nystagmus to the left, vertigo, decreased temperature, pain sensation in the right face and left upper and lower limbs with preserved deep sensation, and difficulty standing and walking. There were no signs of facial palsy, dysarthria, dysphagia, paralysis in the face and four extremities, dysmetria in finger-to-nose and heel-to-shin tests, or urinary symptoms, including UR. Blood tests revealed significantly elevated levels of white blood cells, blood glucose, uric acid, aspartate aminotransferase, alanine aminotransferase, gamma-glutamyltransferase, and C-reactive protein (Table 1). Brain magnetic resonance imaging (MRI) revealed hyperintensities in the right dorsolateral medulla oblongata (Figure 1). He was diagnosed with LMI and treated with antiplatelet medications and rehabilitation therapy.

**Table 1:** Blood test results upon admission.

Inspection items	Result	Reference range
White blood cell count	111 × 10^2^/μL	3,300–8,600
Red blood cell count	542 × 10⁴/μL	435–555 × 10⁴
Hemoglobin	15.8 g/dL	13.7–16.8
Blood platelet	27.8 × 10⁴/μL	15.8–34.8
Total protein	8.3 g/dL	6.6–8.1
Albumin	4.9 g/dL	4.1–5.1
Glucose	135 mg/dL	73–109
Hemoglobin A1c	5.90%	4.9–6.0
Blood urea nitrogen	15.0 mg/dL	8.0–20.0
Creatinine	0.89 mg/dL	0.65–1.07
Total bilirubin	0.6 mg/dL	0.4–1.2
Aspartate aminotransferase	42 U/L	13–30
Alanine aminotransferase	118 U/L	7–30
Alkaline phosphatase	105 U/L	38–113
Lactate dehydrogenase	213 U/L	124–222
Gamma-glutamyltransferase	152 U/L	13–64
Creatine phosphokinase	76 U/L	41–153
Creatine phosphokinase	244 U/L	59–248
Choline esterase	273 U/L	240–486
Amylase	71 U/L	44–132
Sodium	140 mmol/L	138–145
Potassium	4.4 mmol/L	3.6–4.8
Chlorine	101 mmol/L	101–108
calcium	10.1 mg/dL	8.8–10.1
C-reactive protein	0.53 mg/dL	0.00–0.14
Triglyceride	80 mg/dL	40–149
High-density lipoprotein cholesterol	44 mg/dL	40–90
Low-density lipoprotein cholesterol	136 mg/dL	65–139

Following admission, the patient developed dysphagia and UR on day two, necessitating intermittent catheterization. Regarding the UR, the patient reported that the urge to urinate was retained. On day three, Horner syndrome manifested, characterized by right-sided hypohidrosis and ptosis. Swallowing training was initiated on day five, followed by gait training on day 10. During standing and walking training, the patient reported a right-sided BL. By day 20, both vertigo and UR had resolved, and intermittent catheterization was discontinued. By day 40, the patient’s BL had improved, and he achieved independent ambulation. He was discharged on day 70 with independent activities of daily living, although a slight sensory disturbance persisted. A neurological examination one month after discharge revealed no nystagmus, a normal vestibular-ocular reflex, no paralysis, normal facial sensation but reduced superficial sensation (7/10), a normal gait, and no BL. He was also able to return to work.

## Discussion

To our knowledge, this is the second reported case of LMI presenting with both BL and UR. The patient, while relatively young, had multiple risk factors for vascular disease, including untreated hypertension, dyslipidemia, hyperuricemia, and obesity. The blood test results indicated the presence of lifestyle-related diseases and fatty liver, evidenced by elevated levels of blood glucose, uric acid, aspartate aminotransferase, alanine aminotransferase, and gamma-glutamyltransferase. Although the clinical symptoms developed gradually, they were otherwise typical for LMI, with the notable exceptions of BL and UR. Additionally, brain MRI revealed an acute infarction in the right dorsolateral medulla oblongata, consistent with the diagnosis of LMI. Based on these findings, this case was considered a typical presentation of LMI except for the co-occurrence of BL and UR, which is a relatively rare combination. A comparative analysis between the first reported case and the current case is presented in Table 2 [7]. Apart from the laterality of the lesion, the clinical symptoms were remarkably similar, with the primary difference being the presence or absence of cerebellar symptoms.

**Table 2:** Comparison of symptoms between the previous case and this case. N.D.: no data

Symptoms	Previous case	Present case
Location of the lesion	Left medulla oblongata	Right medulla oblongata
Sensory disturbance in the face (R/L)	N.D.	Right side
Sensory disturbance in the body (R/L)	Right side	Left side
Ataxia	Left side	-
Lateropulsion	Toward the left	Toward the right

BL is a condition characterized by an involuntary tendency to lean or fall to one side of the body, despite the absence of apparent weakness or loss of motor control [4,5,8,9]. Although rare in LMI, BL is associated with potential disruptions in several neural pathways: the dorsal spinocerebellar tract (DSCT), the descending lateral vestibulospinal tract (LVST), the vestibulo-thalamic pathway, the dentatorubrothalamic pathway, and the thalamocortical fascicle [8]. The DSCT and LVST are the pathways most likely affected by medulla oblongata infarctions [8]. While DSCT damage can lead to ataxia, LVST damage typically does not [9]. Thömke et al. noted that BL with limb ataxia often involves the DSCT, whereas BL without limb ataxia points to LVST involvement [5]. In our patient, the absence of cerebellar signs suggests that BL was likely due to LVST dysfunction, while in the first reported case, BL was likely attributed to DSCT dysfunction.

UR is also a rare symptom in LMI, but two studies disclosed that UR can be developed in 16% and 19.4% of patients in LMI [6,10]. In addition, seven of nine patients with LMI accompanied by UR underwent a urodynamic study, which demonstrated detrusor underactivity of the bladder in seven patients [10]. This result is consistent with the urodynamic studies that concluded atonic bladder in the first report, demonstrating BL and UR by LMI [7]. Central UR is hypothesized to result from disruption of the neural pathways involved in micturition. Specifically, impairment within the descending pathway from the pontine micturition center (located in the vicinity of the locus coeruleus in the pontine tegmentum) through the dorsal aspect of the inferior olivary nucleus (between the inferior olive and lateral reticular nucleus) in the medulla oblongata and ultimately to the spinal cord can lead to UR [6,7,11,12]. However, while there are bilaterally innervated descending micturition pathways, it is unclear how unilateral medullary infarction can result in complete UR.

Furthermore, based on the existing literature [11,13,14], we have identified the anatomical locations of the pathways associated with BL or UR within the medulla oblongata. Specifically, LVST originates from the ipsilateral lateral vestibular nucleus. This nucleus is situated in the dorsal medulla oblongata, lateral to the internal nucleus bordering the fourth ventricle. The LVST then descends ipsilaterally, uncrossed, through the ventrolateral aspect of the ventral funiculus of the spinal cord [14]. DSCT, in contrast, ascends along the lateral surface of the lower medulla oblongata, positioned immediately anterior to the spinal trigeminal tract, which is located in the dorsolateral medulla oblongata [13]. Additionally, the central micturition pathway has been localized to the dorsal aspect of the inferior olivary nucleus within the medulla oblongata [11]. Given their proximity in the dorsal medulla oblongata, the LVST, DSCT, and the central micturition pathway can be damaged by lesions caused by LMI. This vulnerability may explain the simultaneous occurrence of BL and UR observed following such infarctions.

This report has several limitations. First, it lacked a quantitative assessment of the patient’s recovery process. We were unable to score the patient’s response to drug and rehabilitation treatments. Additionally, a urodynamic objective evaluation of UR would have been beneficial. With a more comprehensive evaluation, including these aspects, we would have been able to provide a more robust presentation of the case.

## Conclusions

This case highlights the rare co-occurrence of BL and UR in LMI. Our analysis of the underlying pathophysiology suggests that BL can arise from the involvement of pathways such as DSCT and LVST, while UR can result from disruption of the descending pathway originating from the pontine micturition center. Importantly, these pathways are located in close proximity to each other within the medulla oblongata, specifically on the dorsal aspect of the inferior olivary nucleus. Therefore, when a relatively large infarction occurs due to LMI, it can concurrently affect these closely situated pathways, leading to the simultaneous development of BL and UR, in addition to typical LMI symptoms such as dizziness and sensory impairment. Understanding the spatial relationship and vulnerability of these pathways provides valuable insight into the complex presentation of symptoms in LMI and underscores the need for comprehensive diagnostic and therapeutic strategies.
